# PRISM: A unified platform for phage isolation and characterization from single-droplet microenvironments

**DOI:** 10.1126/sciadv.aeb2362

**Published:** 2026-03-25

**Authors:** Han Zhang, Justin Boeckman, Rohit Gupte, Ashlee Prejean, Jonathan Miller, Alexandra Rodier, Paul de Figueiredo, Mei Liu, Jason Gill, Arum Han

**Affiliations:** ^1^ Department of Electrical and Computer Engineering, Texas A&M University, College Station, TX 77843, USA.; ^2^ Center for Phage Technology, Texas A&M University, College Station, TX 77843, USA.; ^3^ Department of Biochemistry and Biophysics, Texas A&M University, College Station, TX 77843, USA.; ^4^ Department of Biomedical Engineering, Texas A&M University, College Station, TX 77843, USA.; ^5^ Christopher S. Bond Life Science Center, University of Missouri, Columbia, MO 65211, USA.; ^6^ Department of Molecular Microbiology and Immunology, School of Medicine, University of Missouri, Columbia, MO 65212, USA.; ^7^ Department of Veterinary Pathobiology, University of Missouri, Columbia, MO 65211, USA.; ^8^ Department of Chemical and Biomedical Engineering, University of Missouri, Columbia, MO 65211, USA.; ^9^ Department of Animal Science, Texas A&M University, College Station, TX 77843, USA.; ^10^ Department of Chemical Engineering, Texas A&M University, College Station, TX 77843, USA.

## Abstract

The exploration of bacteriophage diversity remains constrained by reliance on conventional plaque assays, which bias discovery toward phages that form visible plaques. We present PRISM (Phage Recovery and Investigation in Single-droplet Microenvironments), a droplet microfluidics–based, plating-independent platform for high-throughput isolation, quantification, and characterization of bacteriophages. By encapsulating phage-host interactions in water-in-oil microdroplets at single-phage resolution and coupling them with fluorescence-based detection and sorting, we demonstrate PRISM’s capabilities across several key applications: (i) recovery of both “plaquing” and “nonplaquing” *Salmonella* phages from environmental samples, where nonplaquing phages were missed by conventional approaches; (ii) accurate titering of poor-plaquing phages, such as *Rhodococcus* phage ReqiDocB7; and (iii) plating-independent determination of phage resistance and lysogeny frequency, with results closely matching conventional estimates. This highlights PRISM’s enhanced resolution and accuracy, as well as its ability to detect nonplaquing or slow-replicating phages, underscoring the platform’s power to access viral populations overlooked using conventional methods.

## Teaser

A droplet microfluidics platform for studying bacteriophages, a tool for exploring overlooked populations in the virosphere.

## INTRODUCTION

Bacteriophages, or phages, are viruses that infect bacteria and have gained renewed interest for their therapeutic potential and industrial applications in the postantibiotic era (*1*). Phages are also known to be important players in microbial ecosystems, driving bacterial cell turnover and the global carbon cycle by inducing host cell lysis and redirecting nutrients (*2*, *3*). The advent of modern sequencing technologies, such as whole-genome sequencing and metagenomic sequencing, have profoundly impacted phage research and led to a substantial surge in the identification of new phage genomes. These advances also revealed extensive phage diversity and a large reservoir of uncharacterized genes (*4*). However, most phages identified through metagenomics remain uncultured due to limitations imposed by traditional phage culture methods (*5*) and the absence of an approach that enables rapid and high-throughput isolation and amplification of phages.

**Fig. 1.**
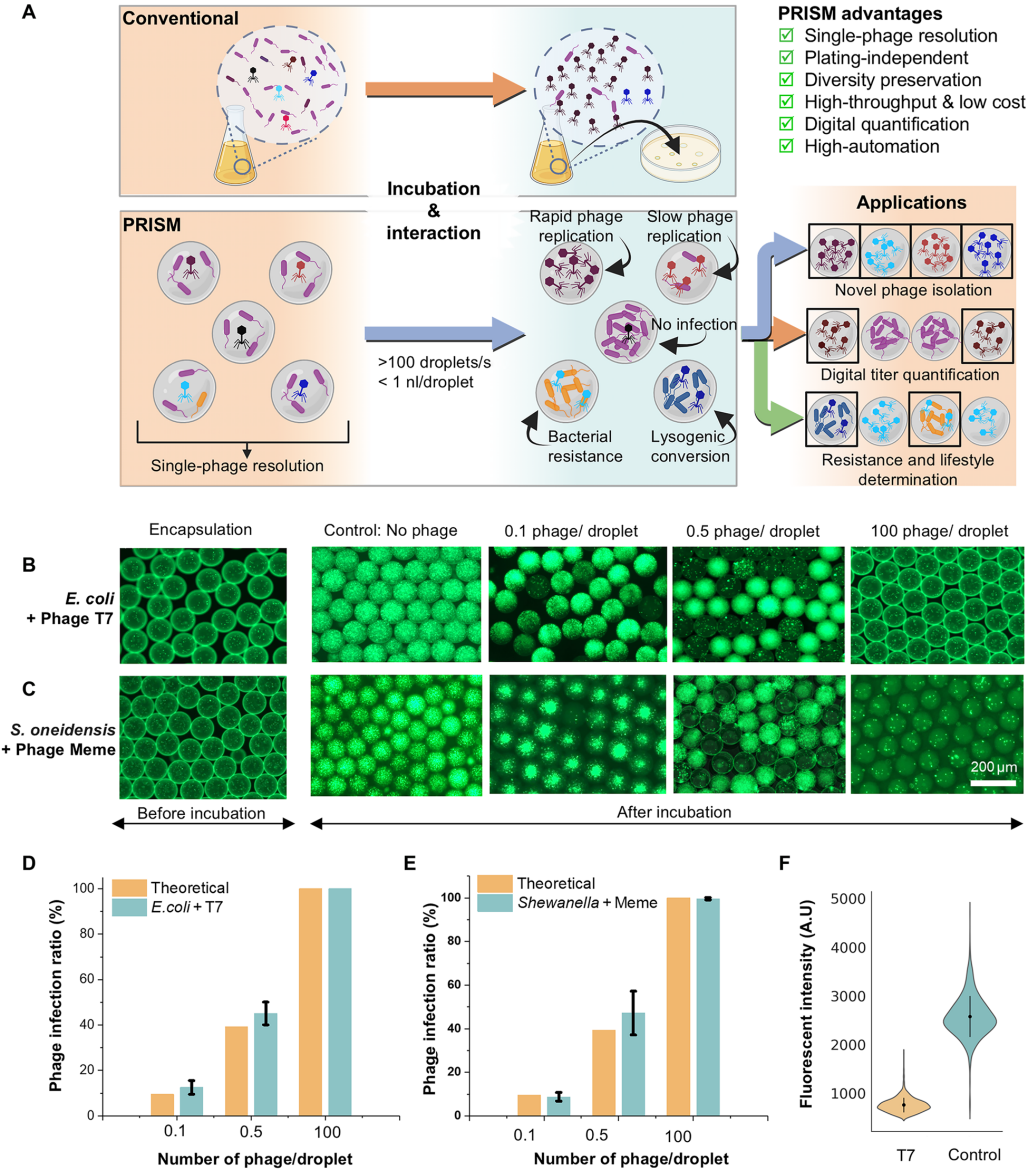
Workflow of PRISM. (**A**) Comparison of conventional plaque assay and the droplet microfluidics–based PRISM workflow. (**B** and **C**) Bacterial killing in droplets by species-specific phages: T7 for *E. coli* and Meme for *S. oneidensis*, tested at three phage concentrations (phages per droplet). Here, 10 to 20 bacterial cells were initially encapsulated in the droplets. (**D** and **E**) Observed infection ratios for *E. coli* + T7 (D) and *S. oneidensis* + Meme (E) align with the expected Poisson distribution of phage encapsulation, supporting single-phage resolution (*n* = 3). (**F**) Fluorescence intensity of *E. coli* droplets with and without T7 phage; lower intensity indicates bacterial inhibition, enabling sorting of active (low-intensity) versus inactive (high-intensity) phage-containing droplets.

Methods for isolating and characterizing phages from the environment have remained largely unchanged for over 100 years (*6*, *7*). Conventional phage isolation methods have several limitations (Fig. 1A, top). The process is time- and labor-intensive, often depending on liquid enrichment and multiple rounds of single-plaque isolation on soft agar overlays for phage purification (*8*, *9*). This isolation from enrichments of mixed phage populations is often biased toward phages with an initial higher abundance and/or better propagation characteristics under standard laboratory conditions, which relies on the lytic activity of phages to form zones of clearing, or plaques, on bacterial lawns. This approach, therefore, results in a constrained representation of phage diversity, overlooking phages that are slow to replicate and fails to capture phages that are unable to form visible plaques in soft agar. While biases toward rapidly growing phages can be beneficial for therapeutic applications, poorly propagating phages retain inherent value as they may be reservoirs for novel genetic elements, such as lytic enzymes or anti-CRISPR proteins. Furthermore, while liquid culture–based methods for isolation of phages infecting slow-growing bacteria or hyperthermophiles do exist, their slow growth and the bacterial densities required to visualize lysis present their own challenges (*10*, *11*). These challenges necessitate plating-independent universal phage isolation approaches that enable the capture of single interaction events between host cells and phages and that facilitate the isolation of undiscovered phages through discrete parallel propagation of mixed phage populations.

Following isolation, phages are evaluated for therapeutic or industrial potential based on their ability to lyse host bacteria. An additional consideration for applied use is phage lifestyle. Virulent phages are restricted to rapidly killing hosts through the lytic pathway, while temperate phages can also integrate into the host genome as dormant prophages (lysogeny) (*1*, *7*). Temperate phages are generally avoided in therapy due to rapid formation of lysogenized, phage-insensitive bacteria. The frequency of lysogeny depends on phage-specific factors and can increase with high multiplicity of infection (MOI) (*12*, *13*), or, if MOI independent, remain constant at rates much higher than spontaneous host resistance. Phage lifestyle and resistance frequency are key screening criteria for determining the applied potential of a given phage. However, they are traditionally assessed via plating-dependent agar-based assays that are low throughput and difficult to automate without notable investment in specialized robotic pipetting systems (*13*–*15*).

Droplet microfluidics has been shown to be a powerful tool for microbiology applications, enabling single-cell encapsulation and the rapid screening of hundreds of thousands of individual cell-encapsulated droplets (*16*–*18*). As a complementary and advantageous alternative to traditional methods, we describe here the development and validation of versatile microdroplet-based phage techniques (Fig. 1A), which provide a streamlined, universal, and plating-independent approach for phage biology, including isolation, quantification, lifestyle prediction, and resistant mutant identification. By encapsulating host bacteria and phages into water-in-oil microdroplets at single-phage resolution, numerous spatially distinct “microenrichments” are created, facilitating rapid and clonal phage isolation while preserving diversity and eliminating the need for the optimization of conditions for time-consuming plaque formation. We term this technology “PRISM” (Phage Recovery and Investigation in Single-droplet Microenvironments). By altering phage-to-host ratios and “hit” droplet criteria, in addition to isolation, we demonstrate that PRISM can also be used for a variety of plating-independent phage characterization applications.

## RESULTS

### Identification of bacterial growth inhibition with single-phage resolution

To demonstrate that phage-mediated host killing can be quantified using PRISM, we used the well-characterized model *Escherichia* phage T7 (Fig. 1B) and the recently isolated *Shewanella oneidensis* phage Meme (Fig. 1C). Various concentrations of phage were combined with exponentially growing host cells and a fluorescent cell viability dye SynaptoGreen C4 (Biotium) (for quantification of live cells in droplets) immediately before droplet generation. After incubation, microdroplets were screened for bacterial growth based on fluorescence intensity and confirmed by direct visualization under bright-field and fluorescent microscopy. High fluorescence intensity indicates bacterial growth and the absence of host cell–affecting phage in a droplet (Fig. 1, B and C). Conversely, low fluorescence intensity indicates low bacterial growth, signifying the presence of phage that inhibited growth of the target host within the droplet. With approximately 0.1, 0.5, and 100 T7 phage particles per droplet [diluted using plaque-forming unit (PFU)/ml titer determined by conventional plaque assay], the percentage of low-intensity droplets was, as expected, 12.5 ± 2.9 (SD), 45.8 ± 5.1 (SD), and 100 ± 0 (SD), respectively. Similarly, when phage Meme was similarly tested at three different phage concentrations (Fig. 1D), the corresponding percentages of low-intensity droplets were 8.8 ± 2.0 (SD), 47.2 ± 9.8 (SD), and 99.6 ± 0.004 (SD), respectively. In both cases, approximately 10 to 20 bacterial cells were initially encapsulated per droplet (fig. S1). These percentages were close to the expected values based on Poisson distribution (*19*). Increasing the phage concentration to approximately 100 phages per droplet resulted in nearly 100% growth inhibition, as indicated by uniformly low fluorescence intensity across droplets (Fig. 1, D and E). These results confirm that PRISM enables quantitative, single-phage-resolution detection of bacterial growth inhibition in droplets.

Next, to demonstrate the high-throughput screening potential of PRISM, we leveraged the inverse correlation between phage activity and fluorescence to distinguish droplets with and without phage using optical laser signal detection (Fig. 1F). Droplets containing T7 phage showed markedly lower fluorescence intensity (mean intensity = 750 ± 150, *n* = *1426*) due to inhibited *Escherichia coli* growth, while control droplets (no phage) retained high fluorescence intensity (mean intensity = 2565 ± 429, *n* = 910) from higher numbers of bacteria due to growth. This binary fluorescence readout enabled digital, single-phage-resolution screening of functional phage-host interactions at rates greater than 100 droplets/s.

### PRISM isolation of plaquing and nonplaquing phages

**Fig. 2.**
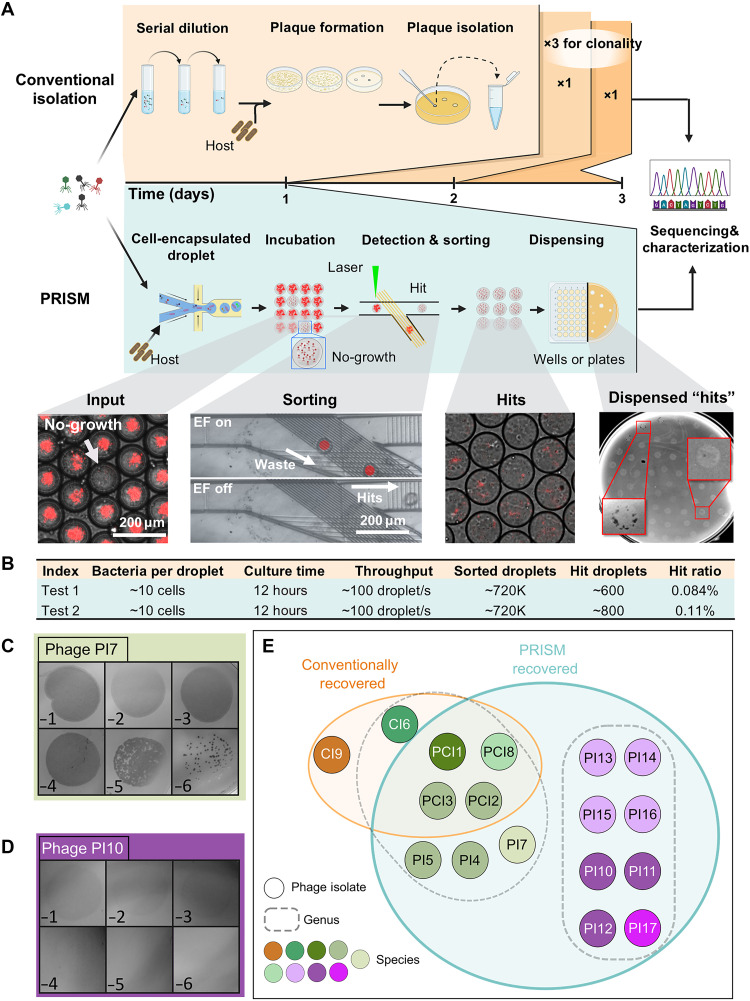
PRISM enables high-throughput screening and recovery of both plaquing and nonplaquing *Salmonella* phages. (**A**) Schematic comparison of conventional agar plate-based phage isolation versus PRISM-based droplet microfluidics workflow. Red boxes highlight plaques or clear zones. (**B**) Summary of PRISM-based screening assay results. (**C** and **D**) Spot assays showing 10-fold serial dilutions of recovered phages, demonstrating both (C) plaquing and (D) nonplaquing phage phenotypes. (**E**) Venn diagram representing recovered phages, taxonomically clustered based on genome alignment of sequenced phages using progressiveMauve.

To demonstrate the utility of PRISM, we isolated phages from environmental samples infecting a clinically relevant strain of *Salmonella enterica* serovar Typhimurium (*S. Typhimurium* ATCC 53648) that constitutively expresses red fluorescent protein (RFP). In brief, filter-sterilized wastewater samples were pooled and virions were concentrated via polyethylene glycol (PEG) precipitation. This concentration process using physical and chemical methodologies was selected to preserve phage diversity and avoid “winner-take-all” phage-host propagation dynamics (*6*, *20*, *21*). Phage-encapsulated microdroplets generated from this sample were incubated overnight before laser-based detection and sorting for droplets in which phage-mediated growth inhibition of host cells (*S. Typhimurium*) could be detected (Fig. 2A). Notably, the titer of the concentrated environmental sample was first determined via top-agar overlay technique, and input phage concentration was kept sufficiently low such that each microdroplet contained no more than a single phage particle. Single-phage encapsulation was further supported by the hit ratio, which was less than 1%, and thus based on the Poisson distribution this means that the presence of multiple phages in a single encapsulation event would be highly unlikely at this level of input. Representative droplet images of key steps in the PRISM workflow are shown in Fig. 2A, including (1) rare droplets in which phage inhibited bacterial cell growth (RFP-dark) surrounded by droplets containing high densities of bacteria (RFP-bright) in the initial library (Fig. 2A, input), (2) sorted droplets enriched for phage that inhibited bacteria growth (Fig. 2A, sorting & hits), and (3) sorted droplets dispensed onto top-agar overlays for infectivity validation. Infectivity was confirmed by the presence of plaque formation and/or zones of clearance from the dispensed RFP-dark droplets on *Salmonella* top-agar lawns (Fig. 2A, dispensing). In parallel, to benchmark our findings we performed phage hunts using conventional approaches from the same PEG-concentrated sample (Fig. 2A, top). Using genetic sequences of these conventionally isolated phages, polymerase chain reaction (PCR) primers were designed to de-duplicate PRISM-identified phages. Unique PRISM phage isolates were then sequenced. This workflow was repeated twice using the same PEG-concentrated sample, thereby providing opportunities for assessing the reproducibility of the system. The tests yielded a phage hit ratio of 0.084 to 0.11%, with corresponding statistics summarized in Fig. 2B. The droplet sorting process during the PRISM assay is shown in movie S1, and representative examples of the confirmation of PRISM dispensed plaques/zones of clearing are shown in fig. S2.

The sorted hit droplets from test #2 were then closely examined. A total of 80 regions in the *Salmonella* top-agar lawns showed evidence of possible phage activity (Fig. 2A, dispensing). Of these, 24 regions were capable of further phage propagation. PCR analysis identified 11 of the isolates as matches to conventionally recovered phages. The remaining 13 were uniquely recovered by PRISM. Of the 13, 11 were successfully sequenced for further analysis. Notably, PRISM-recovered phages fell into two distinct phenotypes of plaque morphology. One morphology, represented by phage PI7, was characterized by standard plaquing that produced distinct circular zones of clearing, which resolved into individual plaques upon serial dilution (Fig. 2C). The other phenotype, represented by phage PI10, was characterized by a “nonplaquing” morphology. In this case, high dilutions of phages resulted in only faint turbidity, and further dilution did not yield individual plaques (Fig. 2D). For the remainder of this work, we define the term “nonplaquing” to mean phages that require extensive optimization for visible plaque formation well beyond the standard top-agar overlay technique, those whose zones of clearing or plaques do not dilute in a linear fashion (indicating a majority of individual virions do not form a plaque), and/or dilutions of phage only resulted in faint zones of turbidity without forming individual defined plaques. In the case of PI10, the nonplaquing phenotype is likely due to generally poor infectivity of this host strain; when assayed in microtiter plate assays or PRISM, growth of the *Salmonella* host strain was only mildly inhibited. Comparatively, phage PI7 was shown to have a much greater impact on host growth in microtiter plate assays or PRISM, and in both cases, regardless of plaquing ability, neither phage (PI10 nor PI7) was able to completely suppress the rapid growth of the host strain (fig. S3). However, complete growth suppression of the host could still be seen in a fraction of infected droplets, likely enabling their initial isolation using the PRISM system. Movie S2 illustrates PI7-infected *Salmonella* droplets exhibiting three distinct outcomes: no inhibition, partial inhibition, and complete inhibition.

In total, the conventional and PRISM approaches recovered 6 and 15 unique phages, respectively. Eleven of the PRISM-recovered phages were unique to the PRISM approach. Notably, a group of these phages was found to display the nonplaquing phenotype, meaning that they did not form visible plaques under standard top-agar overlay plating parameters, and was presumably the reason that they could not be identified by conventional strategies (Fig. 2E).

### Taxonomic diversity recovered by PRISM versus conventional isolation

Sequencing of the unique phages and genomic comparison indicated that, cumulatively, the PRISM and conventional isolation approaches recovered nine distinct taxonomic species, using 95% average nucleotide identity as a cutoff (*22*). Phages PI13, PI14, PI15, and PI16 represent members of a species within the genus *Chivirus*. Phages PI10, PI11, and PI12 represent another distinct species-level group within the *Chivirus* genus, and phage PI17 represents another novel species within the *Chivirus* genus. Five additional species-level taxa within the genus *Kuttervirus* were also recovered, corresponding to the following clusters: (i) PCI3, PCI2, PI4, and PI5; (ii) PI7; (iii) PCI1; (iv) PCI8; and (v) CI6 (table S2). Phage CI9 represents a species within the genus *Nonanavirus* (table S2). The conventional approach recovered phages representing five of these different species, while PRISM recovered representatives from seven distinct species (Fig. 2E and figs. S4A and S5). Notably, the PRISM-recovered phages included three groups of related nonplaquing species, which were not observed with conventional approaches.

Given the recovery of nonplaquing phages from PRISM, which were missed by the conventional approach, we hypothesized that phages such as these would be more difficult to recover and less frequently recovered by conventional means. While not a perfectly quantitative metric, to support this hypothesis, representative genomes from each nonplaquing and plaquing species were compared to deposited phages in the nr/nt NCBI database (fig. S4A). Given that *Salmonella* phages have been highly studied and are well represented in the NCBI database, we expected that the number of returned hits would be an indicator of biases favoring easily propagatable phages in the database. To include even distantly related phages from the database, a 70% nucleotide identity and query coverage were selected as the cutoffs. As expected, in general, species that were recovered by both the conventional approach and the PRISM approach were more highly represented in the database, returning approximately 110 similar phage hits. Conversely, PRISM-recovered species returned approximately half as many hits, suggesting that they are less frequently recovered.

To explore the genomic structures and similarities of the recovered phages, a representative genome from each species was annotated and compared to view overall synteny and genomic arrangement (fig. S4B). Phages CI6, PCI8, PCI1, PI7, and PC13 were selected as representatives of their respective species within the *Kuttervirus* genus for comparative analysis. These genomes are approximately 157 kb in length and share characteristic features of T4-like phages. They also exhibit 85 to 90% sequence identity with the well-annotated *Salmonella* phage Mooltan (*23*). The species in the *Chivirus* genus, which share the nonplaquing phenotypes, represented by phages PI13, PI10, and PI17, were highly syntenic, having genomes of ~60 kb and ~93% nucleotide similarity to the well-studied model *Salmonella* phage Chi (*24*). Last, phage CI9, the most distinct species-level taxon recovered in this study, is approximately 55 kb in length and ~80% nucleotide identity to *Salmonella* phage 9NA (*25*). Together, these results indicate that PRISM recovered multiple unrelated phage types from a single sample, supporting its capability to recover highly diverse phages.

### Plaque-independent phage titering using PRISM

**Fig. 3.**
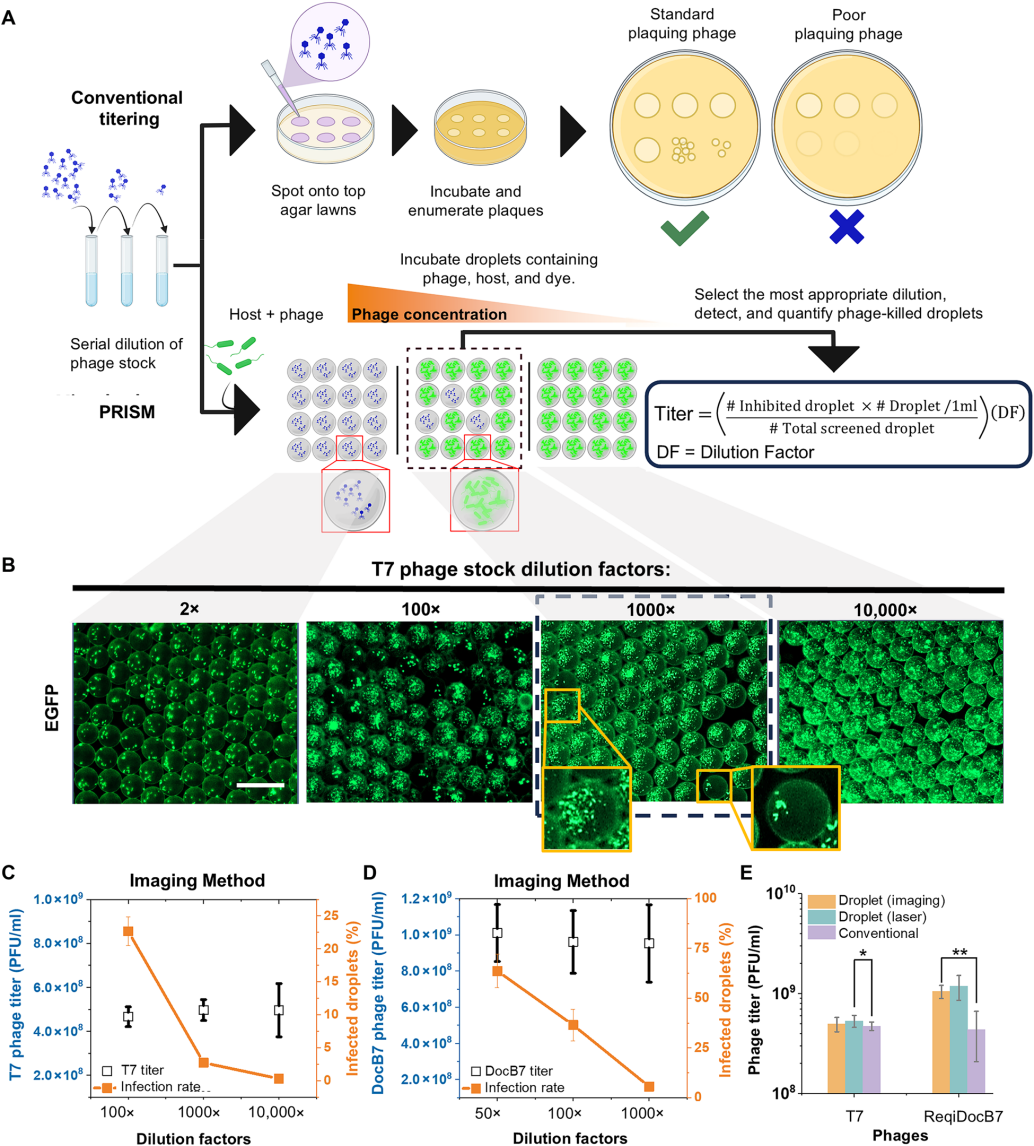
Plating-independent system for high-accuracy phage titering. (**A**) Conventional (top row) and PRISM-based (bottom row) phage titering. (**B**) Phage T7 coencapsulated with *E. coli* at different dilution factors (images were taken after 12-hour incubation; scale bar, 50 μm). (**C** and **D**) Infection rates (orange) and calculated titers (blue) of T7 (C) and ReqiDocB7 (D) phages using fluorescence imaging-based droplet analysis. (**E**) Plaque-forming (T7) and non–plaque-forming (ReqiDocB7) phage titering using PRISM and the conventional method [one-way analysis of variance (ANOVA) test, *P = 0.139; **P = 0.022, *n* = 3].

The original motivation behind PRISM was to broaden the diversity of detectable phages by reducing dependence on plaque formation. The nonplaquing PRISM phages identified in this study were discovered coincidentally. While *Salmonella* species typically support robust and easily countable plaque formation, we have observed that some phage-host systems fail to produce visible plaques reliably, despite extensive optimization efforts. This inconsistency, along with our recovery of nonplaquing PRISM phages, prompted us to explore PRISM as a plaque-independent method of phage quantification (Fig. 3A). In this context, the infection rate was defined as the ratio of total inhibited droplets to total screened droplets.

On the basis of the Poisson distribution, we have the infection rate (droplet containing at least one phage, Eq. 1) and the average number of phages in each droplet (λ, Eq. 2). Phage titer (*T*) can then be calculated by the infection rate, dilution factor, and Poisson distribution as shown in Eq. 3.

$$P\left(k>1\right)=1-P\left(0\right)=1-e^{-\lambda }$$ (1)

λ=Ln(1−Infection rate) (2)

$$\begin{array}{c} T=-\text{Ln}\left(1-\text{Infection rate}\right)\times \text{Dilution factor}\;\times \\ \text{Number of droplets}/\text{mL} \end{array}$$ (3)

To validate the accuracy of the PRISM-based titering method, we first tested it using the well-characterized reliable plaque-forming phage T7 and its *E. coli* host. The infection rate of T7 phages coencapsulated with *E. coli* K12 at different dilution factors was used for detection and phage quantification (Fig. 3B and fig. S7). We directly compared phage titers determined by conventional plaque assays with those obtained using PRISM via both fluorescent microscopy–based droplet imaging and high-throughput laser-based droplet detection methods (Fig. 3, C and E). The droplet imaging method involved taking multiple micrographs and subsequent image processing to identify phage-inhibited hit droplets. Two methods of PRISM-based titering were evaluated to compare their relative accuracy and necessity for high-throughput droplet detection. Regardless of phage dilution factor and subsequent infection rate, the total percentage of killed droplets (Fig. 3C, orange line), was used for titer calculation, yielding similar results. Thus, conventional plaque-based and PRISM-based titering of T7 were largely in agreement. Notably, laser-based droplet detection enabled more convenient (faster readout) and scalable analysis. The higher phage titer determined by PRISM was consistent with trends of plaque-independent liquid-based methods (*26*–*28*), confirming that PRISM can yield reliable and accurate titer measurements.

Having validated our PRISM titering approach, we used an environmental wild-type bacterium *Rhodococcus equi* and the previously isolated phage *Rhodococcus* phage ReqiDocB7 (*29*) to extend this use case. Because of the poor-plaque formation (faint plaques that are difficult to visualize) associated with phage ReqiDocB7 on *R. equi* lawn under standard plating conditions (fig. S6), plaque-based titering of ReqiDocB7 lysates by traditional top-agar overlay cannot be reliably used. Therefore, we sought to establish PRISM’s ability to accurately quantify the titer of ReqiDocB7. Conventional titering estimated ReqiDocB7 to be 3.76 × 10^8^ PFU/ml. However, this method exhibited high variability (as can be seen by the large error bars in Fig. 3E) due to poor plaque formation inherent to the phage and plaque morphology was heavily influenced by plating conditions, such as agar quality and overlay thickness. In contrast, PRISM-based titering using either microscopy- or laser-based droplet detection methods yielded a more consistent result, estimating the titer at approximately 1 × 10^9^ PFU/ml, about twice as high, with significantly reduced variation (Fig. 3E). This finding demonstrates that the PRISM-based detection of phage-inhibited droplets could achieve a more accurate determination of poor plaque-forming phage titer when conventional titer determination is unreliable. Together, these results demonstrate that the PRISM-based approach enables accurate quantification of phages even for phage-host pairs that do not support plaque formation, thereby extending its utility beyond phage isolation.

### PRISM determination of bacterial phage resistance and phage lifestyle

**Fig. 4.**
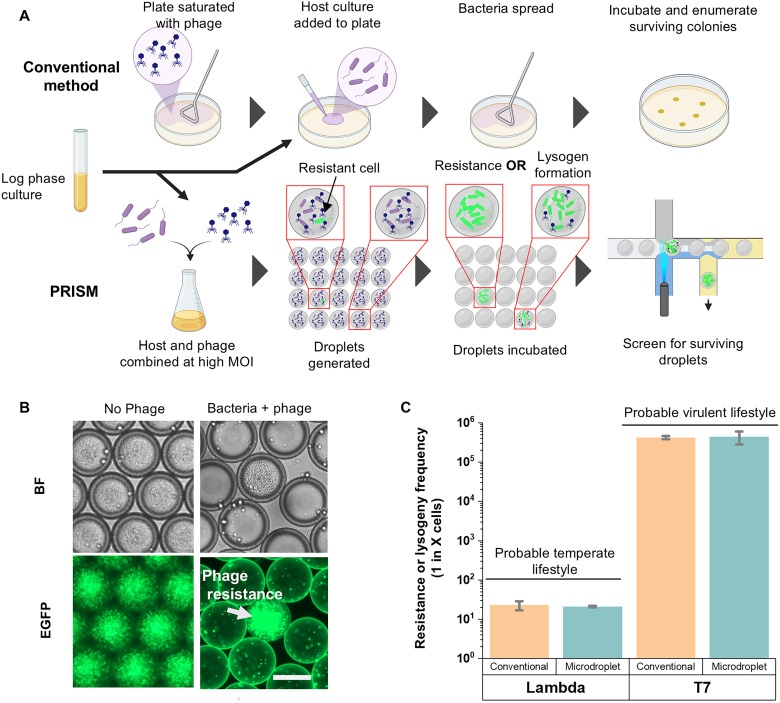
PRISM-based plating-independent system that can determine phage resistance frequency, recover phage-resistant bacterial mutants, and predict phage lifestyle. (**A**) Quantification of phage-resistant mutants using conventional and PRISM methods. (**B**) Example of a rare droplet containing T7 phage-resistant *E. coli* when infected at a high MOI (MOI = 100); scale bar, 50 μm. (**C**) Frequency of resistance to T7 as determined by conventional or PRISM approaches, and determination of lysogen formation in droplets using phage lambda. The discrepancy in the frequency of surviving droplets indicates phage lifestyle.

In addition to phage isolation and quantification, we used PRISM to characterize the phage resistance frequency of bacteria and phage lifestyle (Fig. 4A, bottom). In the conventional approach, a plate is seeded with a high concentration of phage and host culture is spread on top, followed by incubation to identify and enumerate surviving colonies. Surviving colonies become insensitive to phage via bacterial mutations that confer phage resistance (e.g., by loss of the phage receptor), or by formation of lysogens, in which the phage integrates into the host genome and renders it immune to further phage infection. Adapting this concept to PRISM, phages were coencapsulated with the host at a high MOI, incubated, and screened for rare droplets containing high-density bacterial growth, indicating resistance to the applied phage or the formation of lysogens, using laser-based droplet fluorescence detection. This approach enabled resistant variants to be observed in droplets (Fig. 4B). For validation of the phage resistance or lysogenization frequency determination, we used the well-characterized *E. coli* phage T7 (lytic phage lifestyle) and lambda (temperate phage lifestyle). By quantifying the number of droplets showing robust host cell growth, the total number of droplets screened, and the starting number of bacterial cells per droplet, the frequency of phage resistance in the population was determined. For comparison, an aliquot of the same culture was simultaneously assayed for resistance frequency by conventional means (*14*, *15*) (Fig. 4A, top). The frequency of phage resistance for phage T7 determined by PRISM was one in 4.4 × 10^5^ bacterial cells and, in support of this value, the conventional approach estimated resistance frequency to be one in 4.2 × 10^5^ bacterial cells (Fig. 4C). These values were also consistent with the reported frequency of phage T7 resistance (*30*).

In addition to phage resistance frequency, we tested the hypothesis that PRISM enables rapid prediction of temperate and virulent phage lifestyles, which are key features for therapeutic phage selection (*1*, *7*). Using the model temperate phage lambda, lysogenized cells were recovered at rates corresponding to one in 23 cells when using the conventional method and one in 21 cells when using the PRISM approach (Fig. 4C). While the lysis-lysogeny decision remains incompletely understood for many temperate phages, studies have shown that higher MOI often correlates with increased rates of lysogeny for several well-characterized phage repressor systems (*13*). In other cases, lysogeny occurs at a relatively constant rate that is significantly higher than the frequency of mutational resistance (*12*). Therefore, it is expected that temperate phages assayed at high MOI (MOI >1) in the PRISM system will always produce surviving lysogenized droplets at a frequency considerably greater than that of the mutational resistance frequency to a phage, allowing identification of temperate phages (Fig. 4C). Together, these findings demonstrate that in addition to phage isolation, the PRISM system has additional utility for phage characterization in a plating-independent manner.

## DISCUSSION

While recent studies have begun investigating phage isolation using microfluidic platforms (*31*, *32*), PRISM represents the first system for efficient phage isolation and characterization. Furthermore, benchmarking of PRISM to conventional approaches from the same input sample established working parameters for the developed technology. PRISM has the potential to streamline phage isolation and characterization workflows by removing the need for time-consuming subculturing to ensure clonality, shortening the overall process by multiple days. Phages could be isolated in under 24 hours, at least 3× faster than traditional methods, and with significantly reduced labor and materials cost.

While the PRISM system offers many advantages, it is not without limitations. Reduced labor and material costs only come after an initial startup cost of required microfluidic instruments and set up; however, with the growing utility and application of microfluidic systems, such instruments could be multiuse, mitigating initial cost concerns. While robotic agar-based platforms can enable automation of plate-based assays, as demonstrated in large-scale robotic screening workflows, such approaches typically require a significantly higher capital investment and remain constrained by plating-dependent formats (*33*, *34*). As a result, flexibility in assay customization and scalability for parallel screening of diverse environmental isolates are more limited compared to the droplet-based microfluidic system developed here.

By compartmentalizing phage-host interactions in picoliter-volume water-in-oil droplets and using fluorescence-based droplet detection and sorting, once scaled and automated, PRISM has the potential to accelerate the workflow, reduce material costs, and capture a broader spectrum of phage diversity. Notably, recent absorbance-activated droplet sorting platforms have demonstrated that simple light-emitting diode (LED)–photodiode or optical-fiber–based optical density (OD) readouts are sufficient to achieve high-throughput droplet screening and sorting at kilohertz rates (*35*–*37*). These examples suggest that future implementations of PRISM could be engineered around compact LED/photodiode or other low-cost optical detection modules, further reducing system cost and complexity while preserving throughput.

Furthermore, it is predicted that temperate phages would be largely invisible to our system, particularly when the rate of lysogeny is high or increases with MOI, as has been demonstrated in many model systems (*12*, *13*). In these cases, a single phage encapsulated with ~10 bacterial cells initially results in a relatively low MOI (~0.1). Following phage replication and host cell lysis within the droplet, newly released virions can rapidly reinfect the remaining bacteria, leading to a much higher effective MOI (>10). Under these conditions, lysogeny may be favored for certain temperate phages. If the total number of virions remains insufficient to trigger “lysis from without,” the droplet is therefore likely to survive and exhibit growth of lysogenized cells. Similarly, phages whose infection cycle does not culminate in cell death or lysis, like filamentous phages, are expected to also be difficult to detect. However, in both cases, this limitation could be overcome with time-course monitoring or with droplet sorting for more variable fluorescence intensity, so long as the phages have a measurable effect on growth rate, even if only detectable at early stages of growth.

Last, although the proof-of-concept applications demonstrated here occur under routine laboratory conditions, we also considered how PRISM may perform with slow-growing, thermophilic, psychrophilic, or anaerobic bacteria. Existing microdroplet literature indicates that the platform is broadly compatible across diverse physiological conditions. Fluorinated water-in-oil droplets remain structurally stable for multiday incubations at 37°C and can support slow-growing or anaerobic species (*38*, *39*), with additional evidence showing that bacterial presence can further enhance droplet stability (*40*). Droplet systems also tolerate high-temperature biochemical operations, including thermal cycling up to ~95°C (*41*, *42*) and assays at 50° to 70°C (*43*), suggesting compatibility with thermophiles. Conversely, picoliter droplets exhibit strong supercooling capacity and resist freezing to temperatures as low as −38°C (*44*–*46*), supporting potential use with psychrophiles. For anaerobes, all droplet operations can be performed inside an anaerobic chamber, a configuration already validated for complex gut microbial communities (*38*). Together, the broad results indicate that PRISM can be adapted to slow-growing, thermophilic, psychrophilic, and anaerobic organisms with relatively minor modifications.

The PRISM system demonstrates inherently higher phage-host contact sensitivity compared to conventional agar plate methods (*32*, *47*, *48*). In a confined picoliter-scale droplet, a single phage following Brownian motion dynamics remains within micrometers of its bacterial targets, drastically increasing the likelihood of successful encounter, adsorption, and infection of its host (*32*, *47*), as well as the subsequent detection of these events via fluorescence-based cell enumeration. In contrast, in conventional plaque-based assays, phages must rely on passive diffusion through a semi-solid medium, where spatial separation, limited mobility, and inactivation on cell debris, among other factors, reduce the probability of successful host cell infection. As the eventual formation of a visible plaque requires multiple successive rounds of infection, these limitations of semi-solid media assays are compounded, particularly for phages that do not propagate efficiently or exhibit poor diffusion capabilities. These fundamental differences in liquid versus solid-state encounter dynamics contribute to our observations regarding more accurate estimated titer and phage infectivity. First, droplet-based phage titers tend to be higher than those determined by plaque assays, especially for poor-plaquing or nonplaquing phages, as demonstrated by *Rhodococcus* phage ReqiDocB7 (Fig. 3). This observation is consistent with trends reported by other plaque-independent enumeration approaches, such as the most probable number, which generally report higher titers than plaque assays where PFUs can be considered closer to the minimum number of viable particles in a solution (*26*–*28*). The greater discrepancy between titer values obtained by PRISM and the plaque assay for ReqiDocB7 compared to T7 can likely be attributed to the high adsorption rate and “hyper virulent” nature of T7 (*49*), whose infection dynamics make it better suited for fairly accurate enumeration via plaque assay. These features of T7, as well as those of phage Meme, likely also explain their single-particle infection rates closely approximating the predicted theoretical values (Fig. 1, D and E). The forced spatial proximity and repeated contact opportunities in droplets are more likely to overcome physical and stochastic failure modes common in bulk culture or solid media, leading to improved detection of phage activity.

We successfully isolated multiple previously uncharacterized phages from the environment via PRISM. Many of these PRISM-recovered phages were shown to be nonplaquing under standard phage plating conditions used in this study (Fig. 3), indicating they otherwise would have gone undetected in the absence of our PRISM system. We hypothesize that the amplification of the phage within the microdroplet and the subsequent deposition of hundreds or thousands of clonal phage particles onto the top-agar lawn facilitated disruption of the bacterial lawn and their subsequent isolation. Conversely, the deposition of a single phage particle from a nonplaquing phage would likely have little impact on the bacterial lawn, and as such were not recovered by conventional isolation methods. Despite this outcome, the nonplaquing phages described here had nucleotide similarity to numerous phages deposited in the NCBI database, albeit fewer hits than their plaquing counterparts recovered in this study. Given that plaque formation is dependent on a variety of factors such as phage adsorption rate, host growth rate, and diffusion rates, among other factors (*50*), the isolation of similar phages to the nonplaquing ones described here can be attributed to the use of alternate *Salmonella* strains or altered plating conditions. Nevertheless, this further exemplifies the need for a more universal phage isolation methodology that is less dependent on artefacts affecting plaque formation. Having recovered a greater taxonomic diversity, PRISM therefore has the potential to discover otherwise untapped phage reservoirs which could be sources of novel antimicrobials or uncharacterized proteins of great biomedical value.

Last, we further advanced phage-host interaction characterization by quantifying the frequency of bacterial resistance to phage infection and implementing a rapid and robust approach for predicting phage lifestyle. These two parameters, resistance frequency and lifestyle classification, represent critical determinants in evaluating the therapeutic potential and applicability of newly recovered phages (*7*, *14*, *15*, *51*, *52*). Accurate determination of resistance prevalence enables the assessment of selective pressures imposed by individual phages and lends insight into the durability of their efficacy in clinical or environmental contexts. Simultaneously, rapid lifestyle prediction, differentiating lytic from lysogenic phages, is essential, as only obligately lytic phages are generally desirable for antimicrobial applications due to their ability to efficiently clear bacterial populations without integrating into the host genome and rendering the host insensitive to the phage. Our ability to isolate and analyze libraries of phage-resistant bacterial mutants offers a powerful experimental framework for elucidating bacterial defense mechanisms and identifying molecular targets of resistance. These outcomes, in turn, will facilitate the rational design and optimization of therapeutic phage cocktails with improved efficacy and minimized resistance emergence.

In conclusion, PRISM is a plating-independent, droplet microfluidics–based platform that enables rapid and high-throughput isolation, quantification, and characterization of bacteriophages. In head-to-head comparison using the same input samples, PRISM recovered more unique phages than conventional methods, including several that failed to form plaques on conventional soft-agar overlays but exhibited clear lytic activity. This ability to access rare or underrepresented viral types supports the platform’s potential to recover overlooked populations in the virosphere. The platform further enables accurate measurement of phage titer and resistance frequency in a streamlined, agar-free format. Notably, PRISM outperforms conventional assays in titering poor-plaquing phages, offering higher precision where plaque-based methods may yield only rough estimates. Together, PRISM modernizes phage biology by combining speed, resolution, and universality in a single microfluidic system, with ongoing development aimed at expanding its application to diverse hosts and complex microbial samples.

## MATERIALS AND METHODS

### Bacterial strains and phage propagation

*Escherichia coli* (MG1655), *S. enterica* serovar *Typhimurium* (ATCC 53648), and a clinical strain *R. equi* (+103) were cultured in their preferred medium, Luria-Bertani, TSB (tryptic soy broth), and brain heart infusion, respectively, or plated on their respective media supplemented with agar. A chromosomal copy of RFP was previously introduced to *S. Typhimurium*. Previously isolated phages or model bacteriophages were propagated on their respective hosts by the top agar overlay technique (*8*) containing 0.5% agar or 0.25% agar when necessary and supplemented with 5 mM CaCl_2_ and MgCl_2_.

### PRISM chip fabrication

The PRISM droplet microfluidic system fabrication steps are similar to the method published previously (*16*, *18*, *53*, *54*). In brief, a two-photon polymerization instrument was used to fabricate the master mold with sloped structures for the microfluidic device (Nanoscribe Photonics Professional GT, IP-Q photoresist). Electrode patterns were created on the glass substrate by first depositing a Ti/Au layer (20 nm/200 nm) using electron-beam evaporation, followed by photolithography and metal etching. The microfluidic channels were replicated from the master mold in polydimethylsiloxane using conventional soft lithography techniques.

### Generation of phage-bacteria encapsulated droplets

Bacterial cultures were prepared by diluting an overnight saturated culture of 1:500 (v/v) in TSB (Becton, Dickinson) and incubating at 37°C and 250 rpm for 2 hours. The resulting culture was then directly diluted to an OD_600_ of 0.1 (measured using a Nanodrop spectrophotometer, Thermo Fisher Scientific). Fluorescent cell stain SynaptoGreen C4 (Biotium) was added to the bacterial suspension to enable fluorescence enumeration of cells in droplets. When required, phages targeting the bacterial strain were added to the same aqueous phase at varying MOIs, depending on the experimental design.

Phage-bacteria encapsulated water-in-oil emulsion droplets were generated using the PRISM device having a flow-focusing droplet generator geometry (channel cross section: 50 μm × 50 μm). The aqueous phase (bacteria and phage in TSB) was introduced at a flow rate of 300 μl hour^−1^ using a syringe pump (KDS Legto 200), while the continuous oil phase Novec 7500 (3M) containing 2% (w/w) perfluoropolyether-PEG surfactant (PicoSurf 1, Sphere Fluidics) was introduced at 500 μl hour^−1^ using a syringe pump (Legato Systems). All fluids were delivered via Tygon tubing (Saint-Gobain, inner diameter of 0.25 mm) and connected to the PRISM chip immediately after surface treatment with 1% trichloro(1H,1H,2H,2H-perfluorooctyl) silane (Merck) in Novec 7500 for 1 min to minimize droplet adhesion. Droplets (~50 μm in diameter, ~65 pl in volume) encapsulated approximately 10 to 20 bacterial cells each and were produced at a rate of ~100 droplets per second.

### Incubation, droplet counting, and sorting

After generation, droplets were incubated at 37°C in a microfluidic chamber for up to 24 hours to allow for phage infection, bacterial lysis, or continued bacterial growth. In this system, successful phage infection leads to bacterial lysis, while uninfected or phage-resistant bacteria proliferate, producing a strong fluorescent signal within the droplet. A flow-through droplet fluorescence detection setup (fig. S8A) was used to identify and count high-fluorescence hit droplets for laser-based detection. Fluorescence signals were processed using an FPGA I/O data acquisition card (PCIe-7842, National Instruments) and a custom LabVIEW program (fig. S8B). These signals enabled quantification of both total and high-signal droplets for downstream statistical analysis. The detection system was integrated with a feedback-controlled droplet sorting mechanism that activated electrodes to apply dielectrophoretic force to droplets. Correct-sized droplets exceeding a defined fluorescence threshold were selectively sorted (*16*).

### Operation of the PRISM droplet sorting function

All droplet populations were reintroduced into the PRISM droplet sorting device at a flow rate of 35 μl/hour, resulting in a throughput of approximately 30 Hz. The spacing fluid was flowed at 500 μl/hour, with Bias 1 and Bias 2 set to 800 and 1000 μl/hour, respectively. Flow rates were controlled using syringe pumps (KD Scientific Legato series). A 10 kHz, 60 peak-to-peak voltage (Vpp) square wave was applied to the first set of electrodes via a waveform generator (Rigol DG4102). As bright fluorescence droplets pass through the detection region, a feedback loop triggered the second set of electrodes with a 10 kHz, 40 Vpp square wave for 25 ms to perform droplet sorting. The Vpp and sorting duration were adjusted depending on the droplet throughput requirements.

### Conventional and PRISM-based phage isolation

Wastewater samples were collected from three different wastewater treatment plants in the surrounding College Station, Texas area. Samples were centrifuged at 8000*g* for 10 min before being sterilized by passage through a 0.22-μm filter and stored at 4°C until use. To generate a concentrated unenriched phage sample, 60 ml of each sample were pooled and precipitated by addition of PEG-8000 and NaCl to a final concentration of 10% and 1 M, respectively, before overnight incubation at 4°C. This sample was then spun at 10,000*g* for 10 min, and the resulting pellet resuspended in a minimum volume of approximately 1 ml of lambda phage diluent (25 mM tris-HCl, 100 mM NaCl, 8 mM MgSO_4_, and 0.01% gelatin). This concentrated sample was serially diluted and titered via traditional top-agar overlay method to determine the approximate titer of plaque-forming phages. For conventional enrichment-based phage isolation, 10 ml of individual sterile wastewater supernatant was added to 40 ml of TSB and inoculated with 100 μl of an overnight (16 to 18 hour) host culture before being incubated overnight at 37°C with aeration. After overnight incubation, 10 ml of this enrichment culture was centrifuged at 10,000*g* for 10 min at 4°C before being filter-sterilized through a 0.22-μm syringe filter. This enrichment supernatant was then screened on top-agar overlays for plaque formation or zones of clearing. For conventional isolation, morphologically distinct plaques within either enriched samples or PEG-concentrated samples were subcultured for purity and high titer stock lysates generated. For PRISM-based phage isolation, the PEG-concentrated environmental sample was added to TSB containing *Salmonella* host and encapsulated to a target of ~20 cells per droplet. After incubation, phage hit droplets were detected based on lack of RFP signal, sorted, and combined with empty droplets to ensure sufficient distribution of “hits” such that the clonality of phage population within an individual droplet is maintained when deposited onto top-agar overlays seeded with *Salmonella*. Plaques or zones of clearing present in deposited droplet zones were collected for further analysis and/or used for the generation of high titer lysates. Select phages were further characterized via microtiter plate assays, where host was grown to an OD_600_ of 0.5 and then diluted into fresh media, combined with phage to achieve the target MOI. Plates were then incubated with aeration in a Tecan Spark plate reader at 30°C, to slow host growth as needed, or at 37°C and OD_600_ measured every 30 min.

### Droplet dispensing and hit validation

Hit droplets were dispensed using previously published single-droplet-resolution dispensing method (*55*), which uses blank droplets as spacers to isolate and deliver individual hit droplets with high precision. This strategy ensures physical separation between droplets, preventing cross-contamination without the need for complex feedback control. The hit droplets were dispensed onto top-agar overlays seeded with host bacteria of interest. Dispensed hit droplets were allowed to dry before being incubated and inspected for phage activity. Droplets that caused some level of inhibition of the bacterial lawn were collected for downstream sequencing to determine the genotype of the corresponding phage.

### Conventional versus PRISM-isolated phage comparison

To discriminate phages isolated from the PRISM approach that were different than those identified by the conventional approach, conventionally isolated phages were first sequenced and discriminatory PCR primers designed for detection of the conventionally isolated phages (table S1). Pickates recovered from the deposited microdroplets were screened for identity to the conventional phage by plaque PCR using 1 μl of pickate or 1 μl of a 1/10th dilution of pickate into H_2_O as template. Plaque PCR was conducted using 2× Q5 Mastermix with the following thermocycler conditions: 98°C for 10 min, 30 cycles of 98°C for 30 s, 60°C for 20 s, and 72°C for 20 s with a final extension of 72°C for 5 min. PRISM originating pickates generating an amplicon with any of the discriminatory primer sets were determined to likely be a match for one of the conventionally isolated phages, and thus removed from further downstream propagation and analysis. Those pickates that generated no amplicon products for any of the discriminatory primer sets were presumed to represent genetically distinct phages. High-titer lysates were therefore generated, followed by DNA extraction and sequencing as described above.

### Phage DNA and genome analysis

DNA was extracted from high titer phage lysates as described previously using either phenol:chloroform-based extraction or silica DNA spin column-based isolation techniques (*56*). Resulting DNA was prepared for Illumina sequencing using tagmentation based and PCR-based Illumina DNA prep kit, and ultimately sequenced on an Illumina NovaSeq 6000 platform by SeqCenter (Pittsburgh, PA). Resulting reads were assembled and analyzed on the Texas A&M University Center for Phage Technology instance of Galaxy (*57*). After genome sequence completion, genomes were aligned and compared at the nucleotide level by progressiveMauve with DICE similarity index enabled (*58*), species-level clusters validated and determined using Vclust (*59*), and genomes placed taxonomically following ICTV standards using taxMyPhage 3.3.6 (*60*). Phage genomes from a representative of each taxonomic species identified were automatically annotated with Prokka using settings (*61*) with parameters selected for viruses on the European instance of Galaxy (Usegalaxy.eu). Annotated genome maps were generated via EasyFig using the setting tblastX for homology detection. Accession numbers of genomes are available in table S2 with full taxonomic placement.

### Determination of frequency of resistance by conventional and PRISM approaches

Bacterial cultures were grown to early log phase (approximately OD_600_ of 0.2 to 0.4) and the cultures were split to be assayed in parallel by the conventional and PRISM approaches to account for founder effects of phage resistant bacteria. For conventional determination, plate was flooded with 100 μl of high-titer phage lysate at a concentration >10^9^ PFU/ml for a high MOI and allowed to dry in air. Host culture (100 μl) was then applied, incubated overnight at 37°C, followed by enumerating the surviving colonies. In addition, host culture was serial diluted and plated to determine the total CFU/ml of the starting culture. For PRISM, phages were added to host culture at an MOI >10 before droplet encapsulation, incubated, and hit droplets (high fluorescent intensity) detected and sorted. In addition, during the validation of the PRISM approach, sorted hit droplets were dispensed onto agar plates coated with bacteriophage and incubated. At least 10 resulting colonies were subcultured for clonality analysis, and resistance to T7 phage was confirmed by cross-streaking assays. Furthermore, sorted fluorescence-dark droplets were dispensed onto T7-coated plates, which yielded no colonies. To confirm these fluorescence-dark droplets were killed, they were also dispensed onto plates with no phages, which yielded rare colonies; however, these colonies were found to not be resistant to phage T7 and instead represented rare persister cells, which could only grow once phage T7 was removed.
