# Supplementary material for: PRISM: A unified platform for phage isolation and characterization from single-droplet microenvironments
Source: Sci Adv. 2026 Mar 25;12(13):eaeb2362. doi: 10.1126/sciadv.aeb2362 (PMC13015883; doi:10.1126/sciadv.aeb2362)
Supplement: Supplementary file 1 — Figs. S1 to S8 Tables S1 and S2 Legends for movies S1 and S2 [file sciadv.aeb2362_sm.pdf]

Science Advances
AAAS

Supplementary Materials for

PRISM: A unified platform for phage isolation and characterization from single-droplet microenvironments

Han Zhang *et al.*

Corresponding author: Arum Han, arum.han@ece.tamu.edu; Mei Liu, mei.liu@ag.tamu.edu;
Jason Gill, jason.gill@ag.tamu.edu

Sci. Adv. **12**, eaeb2362 (2026)
DOI: 10.1126/sciadv.aeb2362

The PDF file includes:

Figs. S1 to S8
Tables S1 and S2
Legends for movies S1 and S2

Other Supplementary Material for this manuscript includes the following:

Movies S1 and S2

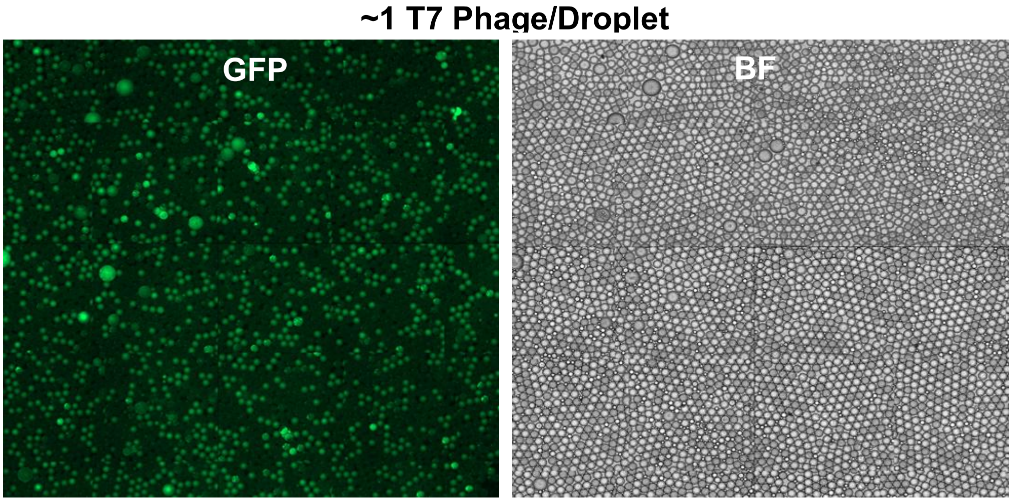

Supplementary Figure 1. Images showing T7 phage infection of *E.coli* at 1 phage particle per droplet.

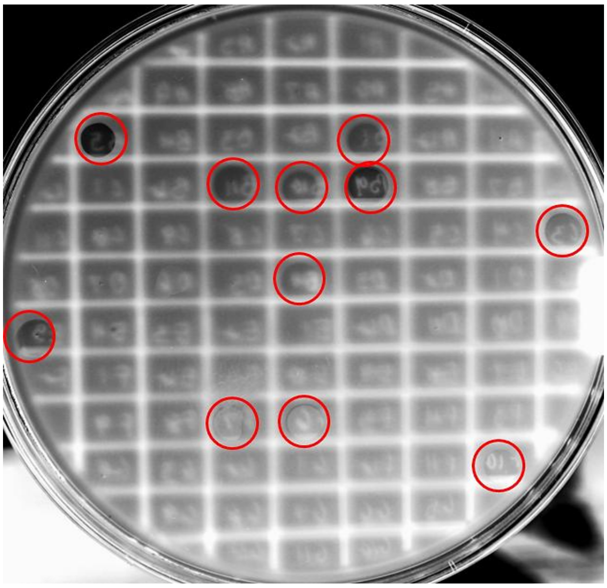

Supplementary Figure 2. Representative confirmation of PRISM plaques following direct dispensing of hit droplets onto top agar overlays. Dispensed zones that displayed evidence of phage activity were picked from the plate and re-spotted for confirmation. Displayed here is a representative confirmation plate, each gridded rectangle represents an area where a putative droplet initially displaying phage activity was spotted for confirmation. Red circles highlight zones with particularly high levels of phage activity, indicated by zones of clearing or plaques.

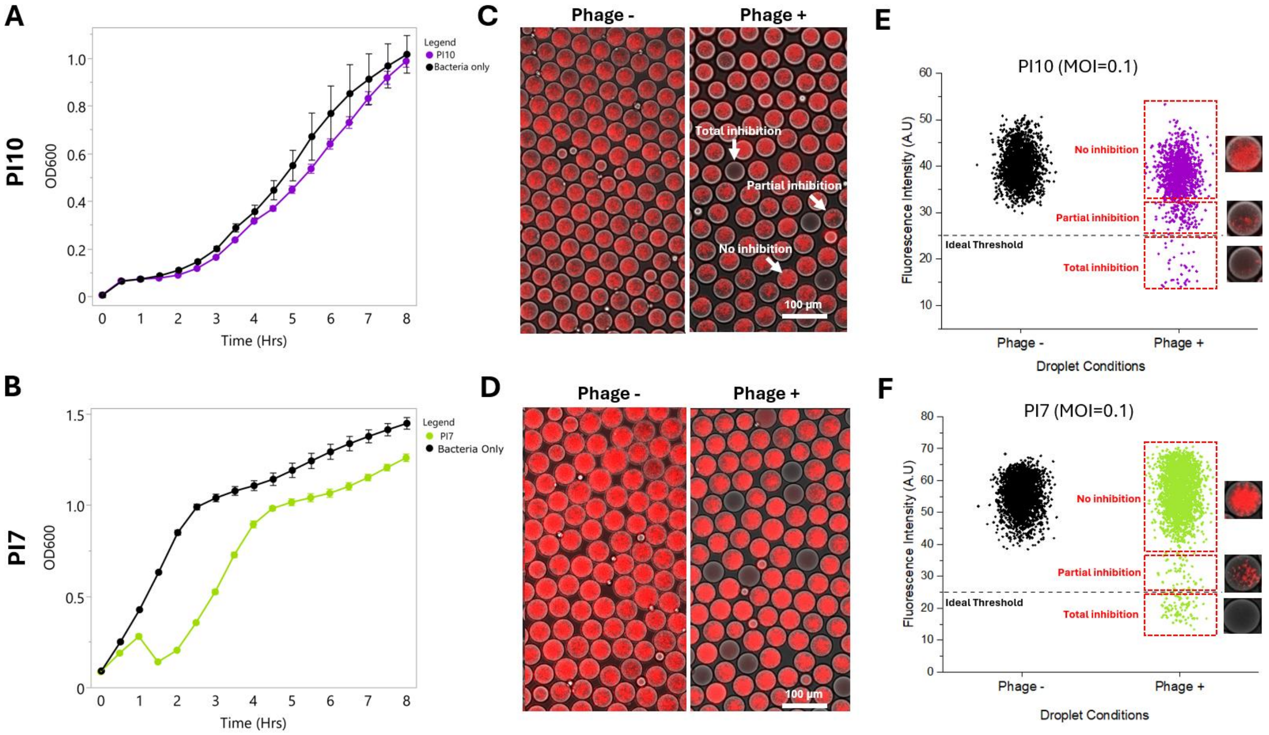

Supplementary Figure 3. Further assays of phages PI7 and PI10 as representatives of plaquing and non-plaquing phenotypes. Microtiter plate lysis assays for (A) PI10 and (B) PI7 at target MOIs of 10 and 1, respectively. PI10 had an extremely mild effect on growth even at high MOI, while PI7 was effectively able to cause lysis and suppress growth briefly. N=3 biological replicates, error bars represent SD from the mean. Representative micrographs of droplets with and without infection by (C) PI10 and (D) PI7 displaying various states of droplet growth inhibition. Fluorescence intensity distribution of droplets infected by (E) PI10 and (F) PI7, displaying total, partial, and no inhibition after 8 h of incubation (N>2000).

(A)

	PCI1	PCI2	PCI3	PI4	PI5	CI6	PI7	PCI8	CI9	PI10	PI11	PI12	PI13	PI14	PI15	PI16	PI17	Relative Novelty #nr/nt hits:
PCI1	100	94.39	92.2	92.08	91.88	87.52	79.59	78.9	1.25	0.07	0.07	0.07	0.07	0.1	0.07	0.07	0.07	110
PCI2	94.39	100	97.7	96.73	97.32	86.33	82.19	81	1.22	0.07	0.07	0.07	0.07	0.1	0.07	0.07	0.07	
PCI3	92.22	97.66	100	98.94	98.7	85.28	82.54	81.71	1.25	0.07	0.07	0.07	0.07	0.1	0.07	0.07	0.07	
PI4	92.08	96.73	98.9	100	98.79	85.23	82.55	81.58	1.25	0.07	0.07	0.07	0.07	0.1	0.07	0.07	0.07	
PI5	91.88	97.32	98.7	98.79	100	85.43	82.71	81.8	1.25	0.07	0.07	0.07	0.07	0.1	0.07	0.07	0.07	111
CI6	87.52	86.33	85.3	85.23	85.43	100	81.51	80.59	1.57	0.07	0.07	0.07	0.07	0.1	0.07	0.07	0.07	111
PI7	79.59	82.19	82.5	82.55	82.71	81.51	100	93.21	1.06	0.07	0.07	0.07	0.07	0.1	0.07	0.07	0.07	111
PCI8	78.9	81	81.7	81.58	81.8	80.59	93.21	100	1.07	0.07	0.07	0.07	0.07	0.1	0.07	0.07	0.07	110
CI9	1.25	1.22	1.25	1.25	1.25	1.57	1.06	1.07	100	0	0	0	0	0	0	0	0	5
PI10	0.07	0.07	0.07	0.07	0.07	0.07	0.07	0.07	0	100	99.76	99.68	92.5	92.49	92.46	91.88	92.22	
PI11	0.07	0.07	0.07	0.07	0.07	0.07	0.07	0.07	0	99.76	100	99.7	92.5	92.49	92.47	91.88	92.22	
PI12	0.07	0.07	0.07	0.07	0.07	0.07	0.07	0.07	0	99.68	99.7	100	92.45	92.44	92.48	91.82	92.23	54
PI13	0.07	0.07	0.07	0.07	0.07	0.07	0.07	0.07	0	92.5	92.5	92.45	100	99.74	99.73	98.47	92.51	
PI14	0.1	0.1	0.1	0.1	0.1	0.1	0.1	0.1	0	92.49	92.49	92.44	99.74	100	99.7	98.46	92.5	
PI15	0.07	0.07	0.07	0.07	0.07	0.07	0.07	0.07	0	92.46	92.47	92.48	99.73	99.7	100	98.42	92.59	
PI16	0.07	0.07	0.07	0.07	0.07	0.07	0.07	0.07	0	91.88	91.88	91.82	98.47	98.46	98.42	100	92.49	54
PI17	0.07	0.07	0.07	0.07	0.07	0.07	0.07	0.07	0	92.22	92.22	92.23	92.51	92.5	92.59	92.49	100	54

Percent Identity
<75
75
85
95
100

Prism Isolated
Conventionally Isolated
Prism+Conventionally Isolated

(B)

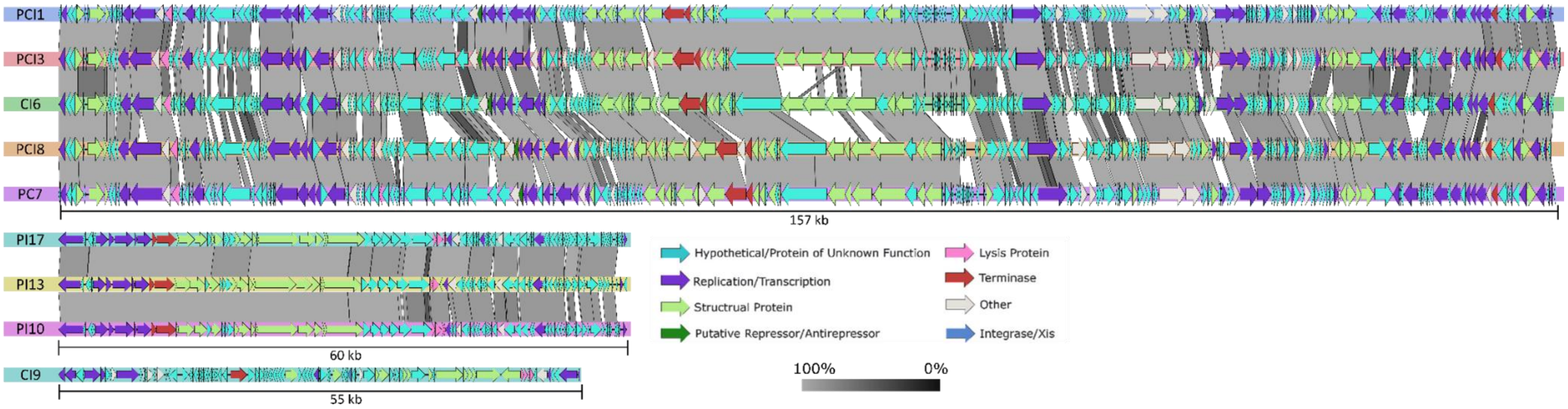

Supplementary Figure 4. Comparison of *Salmonella* phages recovered by both approaches and their relative novelty. (A) Sequenced phage genome assemblies aligned and compared by Progressive Mauve and percent nucleotide identity calculated as DICE coefficient. Returned hits for a representative species isolate using a 70% nucleotide ID and query coverage (Relative Novelty). Legend heat map indicates percent identity. Colored bounding boxes indicate phage clusters within the same species (>95% ID). **(B)** Genome maps and alignment of species representatives identified in this study. Grey bars indicated regions of homology by tblastx indicated by figure legend. General function of proteins indicated by color legend. Species representative color correlates with bounding boxes in **(A)**.

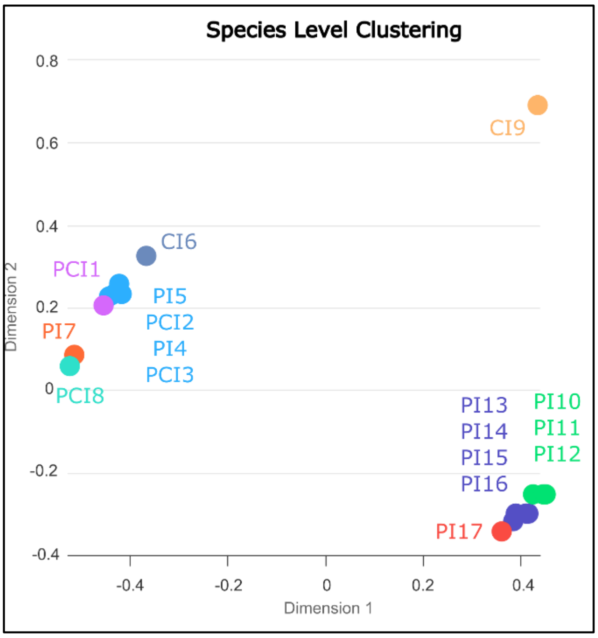

Supplementary Figure 5. Species-level clustering of recovered phages Species-level clustering using Vclust and 95% species level cutoff. Phages occupying a cluster are listed nearby in their respective cluster color, demonstrating the recovery of 9 species level clusters. Complete-linkage algorithm used for genome distances using multidimensional scaling, but displayed illustratively in a 2-D manner.

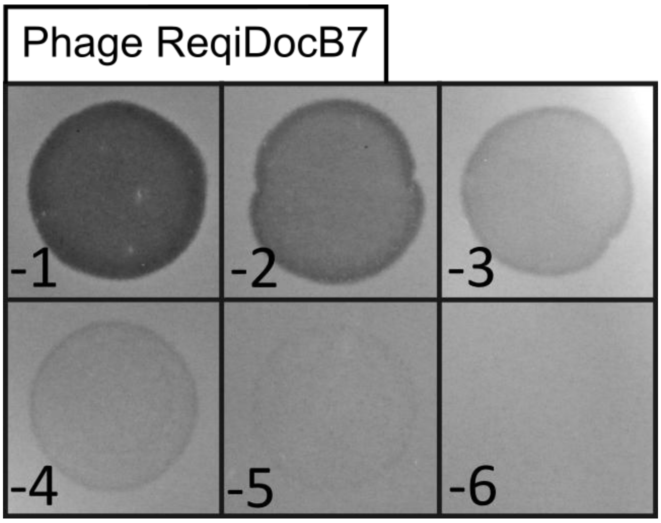

Supplementary Figure 6. Images showing non-plaque forming phenotype of phage RequiDocB7 Ten-fold serial dilutions of phage RequiDocB7 spotted onto lawns of *R. equi* host. Lower dilutions display zones of clearing while higher dilutions display extremely small and difficult to visualize turbid plaques.

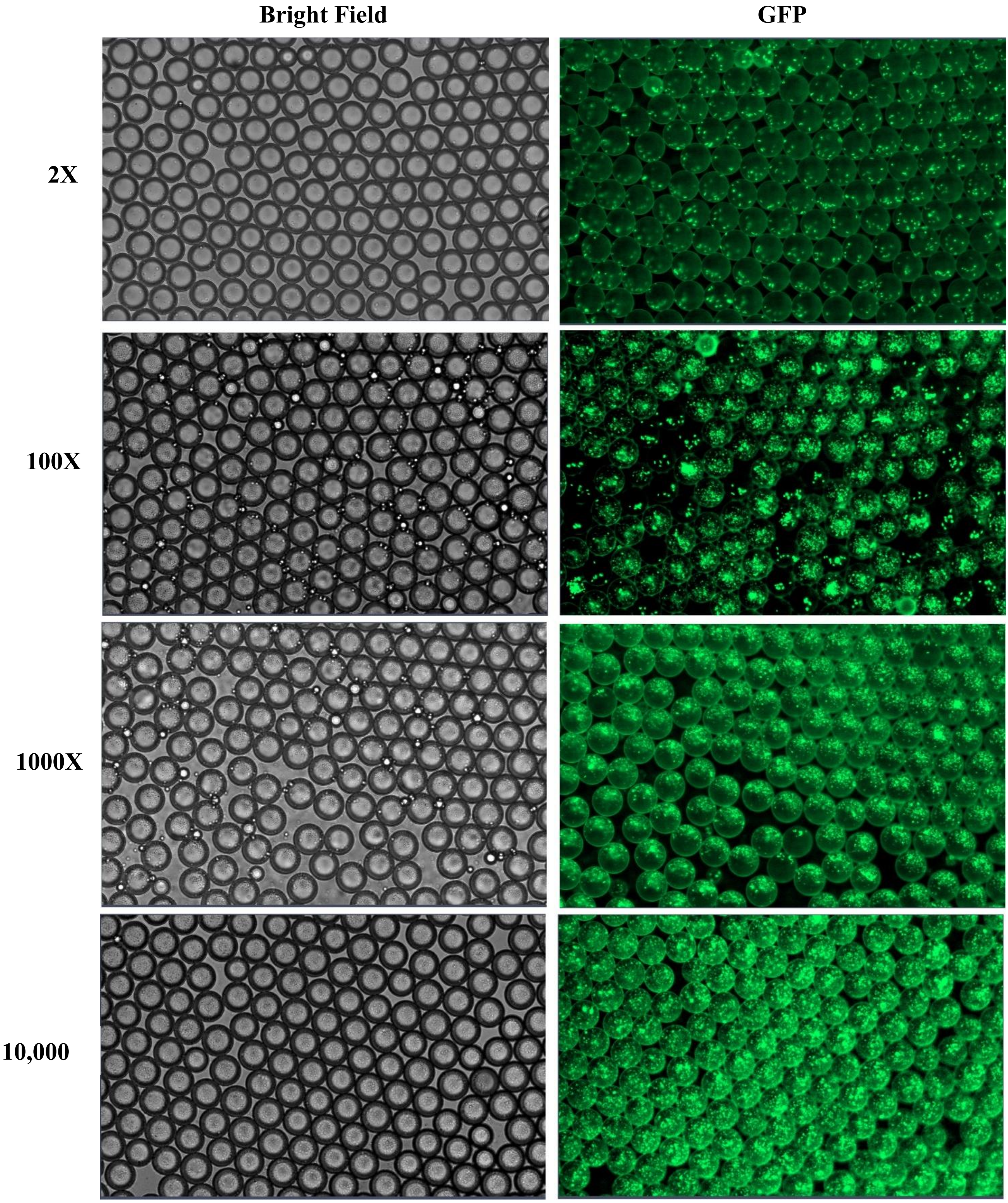

Supplementary Figure 7. The growth inhibition rate of T7-*E.coli* at different dilution factors of T7.

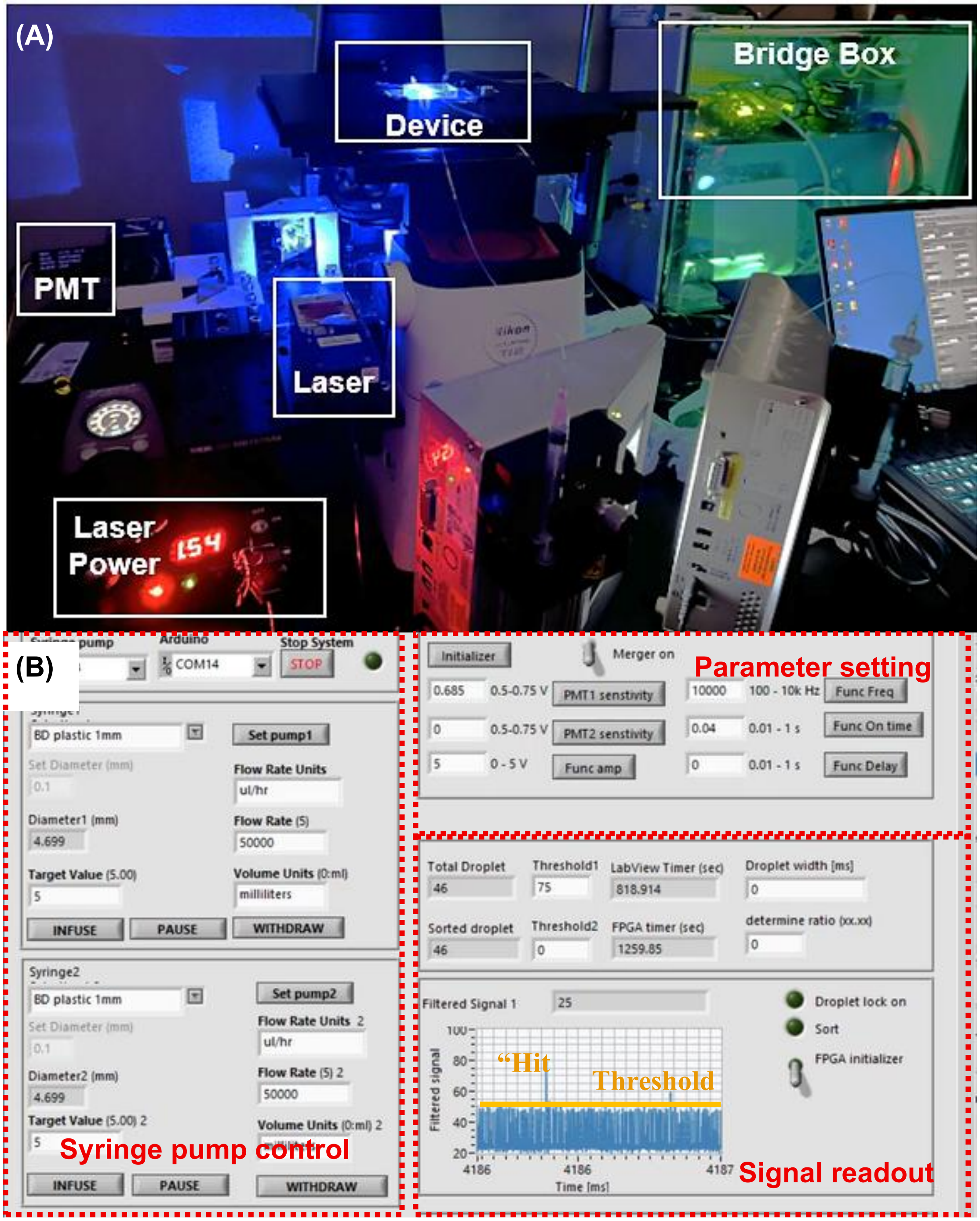

Supplementary Figure 8. The fluorescence-activated droplet sorting platform. (A) Sorting station setup, including the electrical control sub-system (Bridge Box) and the fluorescence detection sub-system (Laser than PMT). (B) LabVIEW™ software user interface used for fluorescence-activated droplet sorting.

Supplementary Table 1. Primers designed based on conventionally isolated phage sequences for deduplication and detection. Original isolation source of conventionally isolated phages, and primer sequences designed for detection and discrimination are summarized.

	Source	Detection Primers F	Detection Primers R
**Phage PCI1**	TAMU Influent Enrichment	5'-CATAATCTTCATGCCACGCC-3'	5'-GGGATTCACTATAAAGACCGC-3'
**Phage PCI3**	Anahuac Influent Enrichment	5'-GTTCGTCGGTCTGGTGATGT-3'	5'-TTCGGAAGCCAGCTGAATTT-3'
**Phage CI6**	Port Arthur Activated Sludge Enrichme	5'-GTTGCTGCCACTTTGCCTG-3'	5'-CCCAAGCGAACTCCAGAGAA-3'
**Phage CI9**	PEG Concentrated Pool	5'-CAATTGTCCCGGCTACCACT-3'	5'-TGAACTGGGTTCACAGGACC-3'
**Phage PCI8**	PEG Concentrated Pool	5'-AATGTCTTTCAGGCGAGGCA-3'	5'-GAAGGCACCATGTGTCCGTA-3'
**Phage PCI2**	PEG Concentrated Pool	5'-TTCCCCTTCCAGTAACCCGA-3'	5'CTGGTGGTGGTATCGTCGTC-3'
**Additional primers for discrimination between phage PCI3 and PCI2:**		5'-TCAGAAGACTCGCCACCAAC-3'	5'-CGAAACGCTGCGATGCAATA-3'

Supplementary Table 2. Phage accession numbers and taxonomy Taxonomic placement of phages as determined by taxMyPhage with default parameters are summarized.

Phage	Accession	Taxonomy
CI6	PX673828	c__Caudoviricetes;o__Pantevenvirales;f__Ackermannviridae;sf__Cvivirinae;g__Kuttervirus;s__novel species within Kuttervirus
PCI8	PX673829	c__Caudoviricetes;o__Pantevenvirales;f__Ackermannviridae;sf__Cvivirinae;g__Kuttervirus;s__novel species within Kuttervirus
PCI2	PX673830	c__Caudoviricetes;o__Pantevenvirales;f__Ackermannviridae;sf__Cvivirinae;g__Kuttervirus;s__novel species within Kuttervirus
PCI1	PX673831	c__Caudoviricetes;o__Pantevenvirales;f__Ackermannviridae;sf__Cvivirinae;g__Kuttervirus;s__novel species within Kuttervirus
PI13	PX673832	c__Caudoviricetes;o__Not Defined Yet;f__Casjensviridae;sf__Not Defined Yet;g__Chivirus;s__novel species within Chivirus
PI15	PX673833	c__Caudoviricetes;o__Not Defined Yet;f__Casjensviridae;sf__Not Defined Yet;g__Chivirus;s__novel species within Chivirus
PI12	PX673834	c__Caudoviricetes;o__Not Defined Yet;f__Casjensviridae;sf__Not Defined Yet;g__Chivirus;s__novel species within Chivirus
PI17	PX673835	c__Caudoviricetes;o__Not Defined Yet;f__Casjensviridae;sf__Not Defined Yet;g__Chivirus;s__novel species within Chivirus
PI14	PX673836	c__Caudoviricetes;o__Not Defined Yet;f__Casjensviridae;sf__Not Defined Yet;g__Chivirus;s__novel species within Chivirus
PI4	PX673837	c__Caudoviricetes;o__Pantevenvirales;f__Ackermannviridae;sf__Cvivirinae;g__Kuttervirus;s__novel species within Kuttervirus
PI10	PX673838	c__Caudoviricetes;o__Not Defined Yet;f__Casjensviridae;sf__Not Defined Yet;g__Chivirus;s__novel species within Chivirus
PI11	PX673839	c__Caudoviricetes;o__Not Defined Yet;f__Casjensviridae;sf__Not Defined Yet;g__Chivirus;s__novel species within Chivirus
PI7	PX673840	c__Caudoviricetes;o__Pantevenvirales;f__Ackermannviridae;sf__Cvivirinae;g__Kuttervirus;s__novel species within Kuttervirus
PI16	PX673841	c__Caudoviricetes;o__Not Defined Yet;f__Casjensviridae;sf__Not Defined Yet;g__Chivirus;s__novel species within Chivirus
PI5	PX673842	c__Caudoviricetes;o__Not Defined Yet;f__Casjensviridae;sf__Not Defined Yet;g__Chivirus;s__novel species within Chivirus
PCI3	PX673843	c__Caudoviricetes;o__Pantevenvirales;f__Ackermannviridae;sf__Cvivirinae;g__Kuttervirus;s__novel species within Kuttervirus
CI9	PX673844	c__Caudoviricetes;o__Not Defined Yet;f__Not Defined Yet;sf__Not Defined Yet;g__Nonanavirus;s__novel species within Nonanavirus

Supplementary Movie S1. Time-lapse visualization of the droplet sorting process during the PRISM assay. Droplets exhibiting inhibited bacterial growth are identified and directed to the "hit" channel.

Supplementary Movie S2. Single-droplet resolution monitoring of phage infection events. PI7-infected Salmonella droplets exhibit three distinct outcomes: no inhibition, partial inhibition, and complete inhibition of bacterial growth.
